# Influenza A viruses alter the stability and antiviral contribution of host E3-ubiquitin ligase Mdm2 during the time-course of infection

**DOI:** 10.1038/s41598-018-22139-6

**Published:** 2018-02-27

**Authors:** Andrés Pizzorno, Julia Dubois, Daniela Machado, Gaëlle Cartet, Aurelien Traversier, Thomas Julien, Bruno Lina, Jean-Christophe Bourdon, Manuel Rosa-Calatrava, Olivier Terrier

**Affiliations:** 1 Virologie et Pathologie Humaine-VirPath team, Centre International de Recherche en Infectiologie (CIRI), INSERM U1111, ENS Lyon, Université Claude Bernard Lyon 1, Université de Lyon, Lyon, CNRS UMR5308 France; 2 Laboratoire des Pathogènes Emergents, Fondation Mérieux. CIRI, UCBL1- INSERM U1111, ENS Lyon, CNRS UMR5308 Lyon, France; 3 Laboratoire de Virologie, Centre National de Référence des virus Influenza, Institut des Agents Infectieux, Groupement Hospitalier Nord, Hospices Civils de Lyon, Lyon, France; 4 Division of Cancer Research, University of Dundee, Ninewells Hospital and Medical School, Dundee, United Kingdom

## Abstract

The interplay between influenza A viruses (IAV) and the p53 pathway has been reported in several studies, highlighting the antiviral contribution of p53. Here, we investigated the impact of IAV on the E3-ubiquitin ligase Mdm2, a major regulator of p53, and observed that IAV targets Mdm2, notably *via* its non-structural protein (NS1), therefore altering Mdm2 stability, p53/Mdm2 interaction and regulatory loop during the time-course of infection. This study also highlights a new antiviral facet of Mdm2 possibly increasing the list of its many p53-independent functions. Altogether, our work contributes to better understand the mechanisms underlining the complex interactions between IAV and the p53 pathway, for which both NS1 and Mdm2 arise as key players.

## Introduction

Influenza viruses are important pathogens responsible for recurrent seasonal epidemics and causing acute febrile respiratory illness^1^. Among the three types of influenza viruses (A, B & C), influenza A viruses (IAV) constitute a serious threat to human populations, with the potential to cause pandemics, as illustrated by the influenza A(H1N1) virus in 2009^1,2^. Influenza viruses rely on numerous host factors and pathways to achieve successful replication. However, despite important progress made throughout the last decade, notably with the contribution of high-throughput “omics’ approaches, many mechanisms underlying influenza-host interactions remain elusive^3–6^.

The transcription factor p53 is well-known for its role as a tumour suppressor, by rapidly accumulating in the cell nucleus in response to stress stimuli, hence modifying gene expression to regulate cell fate. Beyond this major function, p53 is also involved in the regulation of a large panel of biological processes^7,8^. Several studies have investigated the interplay between influenza viruses and different signalling pathways, such as the PI3K/Akt or the NF-κB^9,10^. Interestingly, the functional interactions between influenza viruses and p53 have only been reported in a limited number of studies, mainly focused on the antiviral facet of p53^11–14^. We have previously shown that several levels of regulation of p53 transcriptional activity are affected during the time course of IAV infection, possibly enabling a fine-tuning of p53-mediated biological responses in favour of viral replication^14–16^. We have particularly demonstrated that IAV modulate p53 transcriptional activity, notably through the contribution of viral non-structural protein NS1 to the stabilization of p53 in IAV-infected cells^15^.

Mdm2, considered as the main negative regulator of p53, is an RING-finger E3 ubiquitin ligase that binds to p53 to promote its ubiquitination and degradation^17^. Mdm2 and p53 participate in a negative feedback loop wherein p53 activates the transcription of Mdm2, which in turn inactivates p53 by directly associating with it and promoting its ubiquitination and proteasomal degradation^17^. Since p53 is largely regulated at the post-translational level, the possible alteration of the Mdm2–p53 interaction, decrease of Mdm2 protein levels and/or subcellular delocalisation of Mdm2 have been suggested as the primary mechanisms for stabilization of p53^17^. Nevertheless, only a limited number of studies have investigated the role of Mdm2 in the context of infection by non-oncogenic viruses, such as hantaviruses for example^18^. Although two different genome-wide RNAi studies, both performed in human lung epithelial cells, have highlighted Mdm2 as a crucial factor for IAV infection^5,6^, the role of Mdm2 in IAV infection remains unclear. In that regard, Wang and collaborators have recently shown that the stabilization of p53 in IAV infected cells was associated with a compromised Mdm2-mediated ubiquitination of p53^19^.

The aim of the present study was to further characterise the mechanisms underlying the compromised activity of Mdm2 during IAV infection. Our results indicate that Mdm2 stability is modulated during infection, with a marked degradation at early stages of infection. Different experimental strategies also indicate an antiviral role played by Mdm2, possibly independently of both its E3-ubiquitin ligase activity and p53. Altogether, our results improve our comprehension of the role of Mdm2 during the time course of IAV infection.

## Results

### Mdm2 expression is strongly impacted by IAV infection

**Figure 1:**
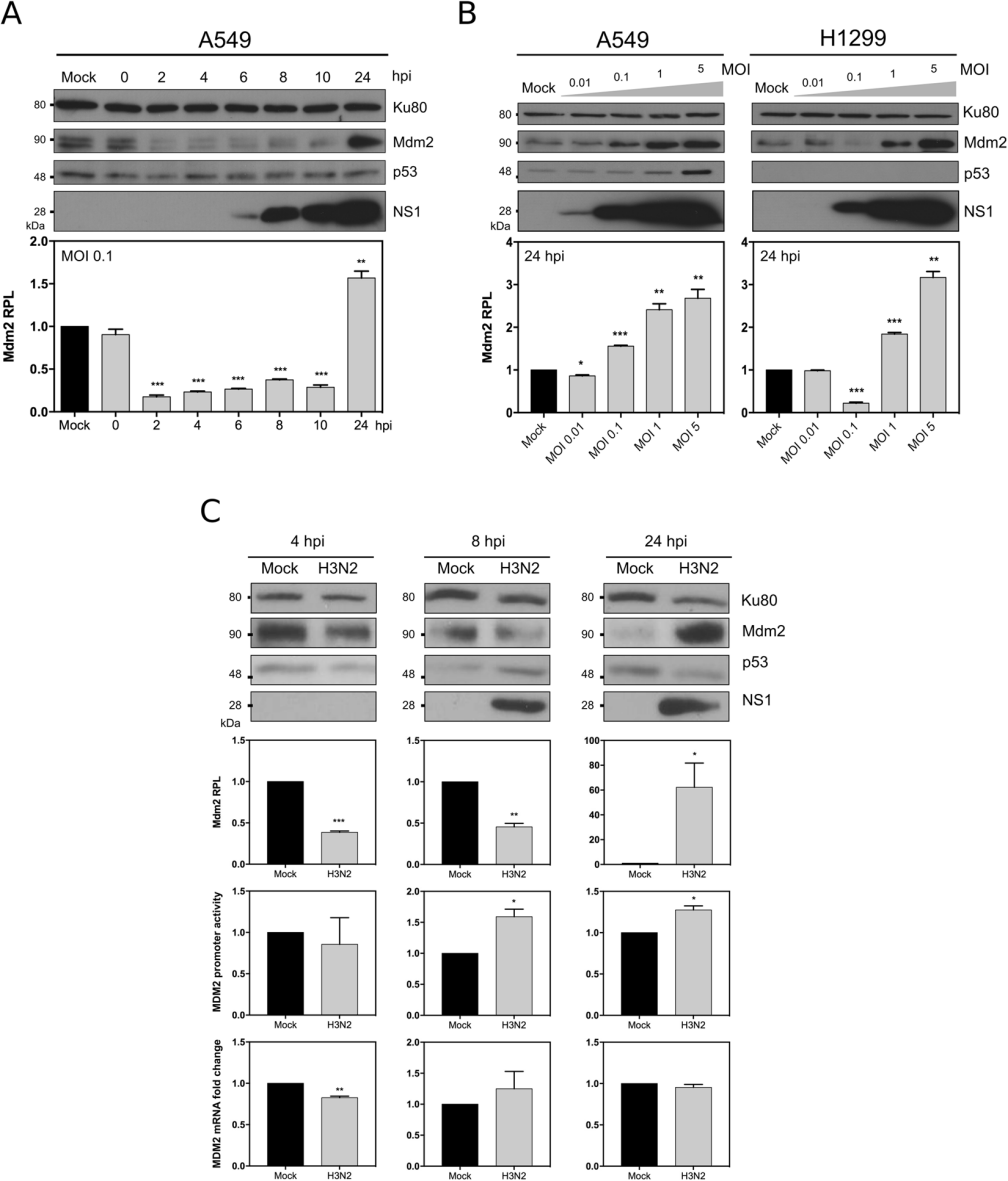
Mdm2 expression is strongly impacted by IAV infection, mostly at post-transcriptional levels. (**A**) Human lung A549 cells were mock-infected or infected by influenza A/Moscow/10/99 (H3N2) at a MOI of 0.1 and cell lysates were analyzed by western blot for the expression of Mdm2 (SMP14 antibody), p53 and IAV NS1 at different time-points post infection. Ku80 was used as a loading control. Mdm2 relative protein levels (Mdm2 RPL) were measured by densitometry and calculated from data from three independent experiments. An asterisk indicates a significant difference compared with the results for mock-infected cells (^**^ and ^***^ for *P*-value < 0.01 and 0.001, respectively). (**B**) Alternatively, A549 or H1299 cells were mock-infected or infected by influenza A/Moscow/10/99 (H3N2) at different MOI and expression of Mdm2, p53 and IAV NS1 were monitored at 24 h post-infection by western blot, using the same approach. (**C**) A549 cells were mock-infected or infected by influenza A/Moscow/10/99 (H3N2) at a MOI of 4 or 0.1 and cell supernatants and lysates were harvested at 4, 8 or 24 hpi, respectively. Endogenous Mdm2 expression was measured at protein level by western blot, Mdm2 promoter activity was monitored using a luciferase reporter plasmid. In addition, Mdm2 mRNA expression was measured by RT-qPCR. An asterisk indicates a significant difference compared with the results for mock-infected cells (^*, **, ***^ for *P*-value < 0.05, 0.01 and < 0.001, respectively).

We firstly investigated the impact of IAV infection on endogenous Mdm2 expression, by performing mock or influenza A (H3N2) infections in A549 human lung epithelial cells (Fig. 1). At a multiplicity of infection (MOI) of 0.1, we observed a dramatic decrease of up to 5-fold in Mdm2 endogenous protein levels at 2 h post-infection (hpi), compared to the mock-infected control. Although Mdm2 relative protein levels (RPL) remained below 50% at least for the following 8 h, a more than 1.5-fold increase compared to mock was observed 24 h after infection (*P* < 0.01, Fig. 1A). Of note, these results were validated using another anti-Mdm2 monoclonal antibody 2 A10 (Supp. Fig. 1), in order to discard any possible detection bias due to potential interference of IAV-induced post-translational modifications of the epitopes recognised by the SMP14 antibody. Moreover, a similar increase in Mdm2 expression at 24 hpi was observed using increasing MOIs in A549 cells (Fig. 1B). Interestingly, this increase was not correlated with a decrease of endogenous p53, suggesting a possible deregulation of p53/Mdm2 loop at late stages of IAV infection. Analogous experiments performed in H1299 cells, which possess a homozygous partial deletion of *TP53* gene, show a similar increase of Mdm2 protein levels at 24 hpi, hence advocating for a p53-independent nature of this phenomenon (Fig. 1B). The p53-independent impact of IAV infection on Mdm2 expression was further confirmed by performing experimental infections an IAV of the H1N1 subtype in both A549 and H1299 cells. Of note, with differences in terms of the extent or kinetics of this modulation were observed, probably due to viral strain/subtype specificity. (Supp. Fig. 2A,B).

To further characterize the impact of infection on Mdm2 expression, we mock-infected or infected A549 cells to reproduce different viral conditions of marked decrease (MOI 4, 4 and 8 hpi) or increase (MOI 0.1, 24 hpi) of Mdm2 RPL compared to mock and measured Mdm2 mRNA levels by RT-qPCR (Fig. 1C). At 4 hpi, Mdm2 mRNA levels in H3N2 infected cells remained comparable to those observed in mock controls at 8 or 24 hpi (Fig. 1C). In a parallel experiment, we also measured the Mdm2 promoter activity, using a luciferase reporter plasmid. Our results indicated a limited, yet statistically significant (*P* < 0.05) increase of promoter activity in IAV-infected cells compared to mock at 8 and 24 hpi. However, this increased activity did not appear to be correlated with Mdm2 mRNA and/or protein levels at the same time-points (Fig. 1C). Altogether, our results indicate that IAV strongly impacts endogenous Mdm2 expression, most probably at post-translational level.

### IAV modulates Mdm2 nucleo-cytoplasmic localization during the time-course of infection

**Figure 2:**
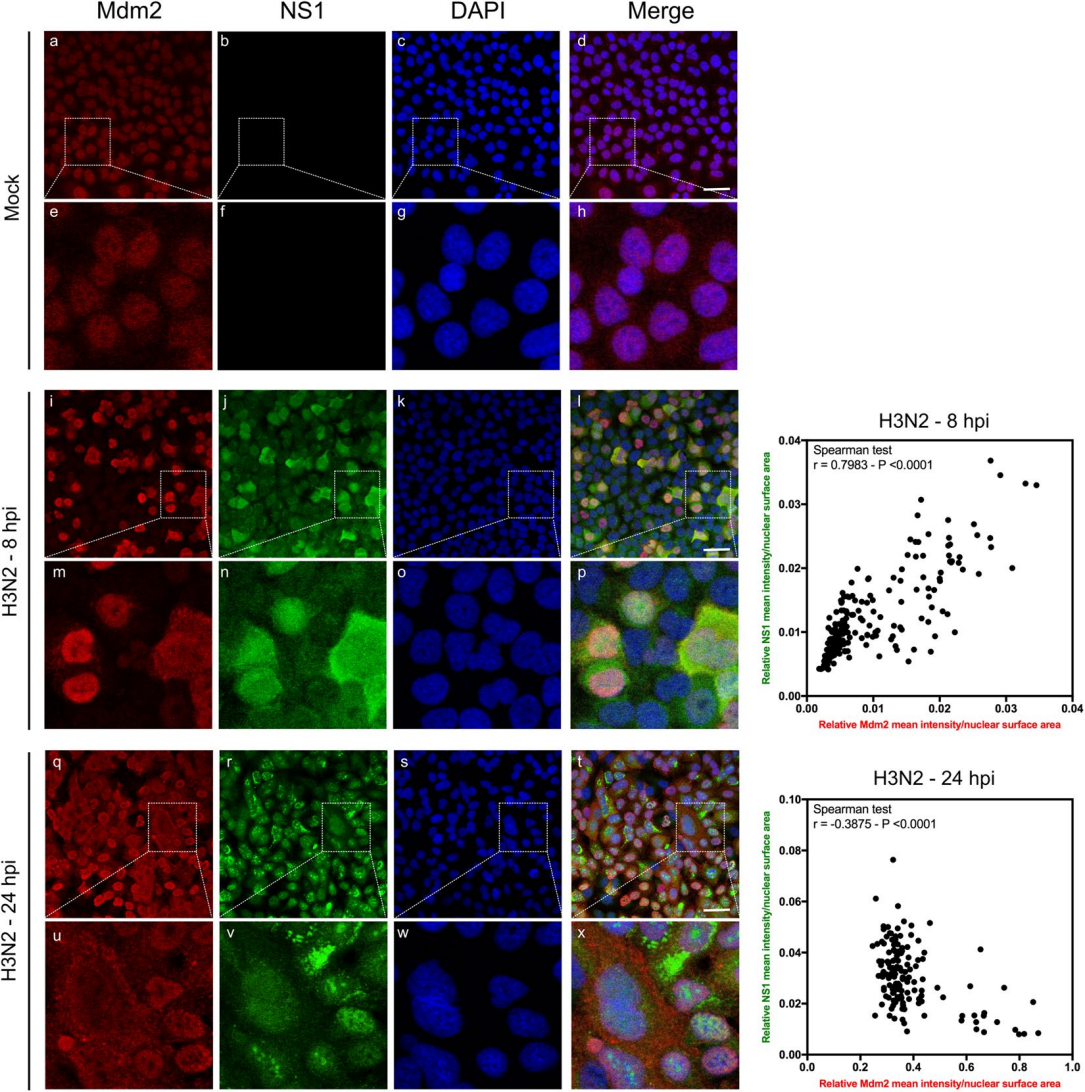
IAV alters Mdm2 protein level and its nucleo-cytoplasmic localization during the time-course of infection. Immunofluorescence staining of Mdm2 (red) and influenza NS1 (green) in mock-infected (panels a to h) or infected with H3N2 virus at a MOI of 1 (panels i to x) was performed at different times, as indicated. Nuclei were counterstained with DAPI (blue, panels c,g,k,o,s,w). Merged fluorescent signals are presented in panels d,h,i,p,t and x. Cell details are enlarged (inset). White scale bar = 10 μm. The presented results obtained by confocal immunofluorescence microscopy are representative fields, from several repeated experiments. The relative mean nuclear intensity of NS1 and Mdm2 stainings was measured at 8 and 24 hpi with ImageJ (version 1.51 h - http://imagej.nih.gov/ij), using DAPI staining to define nuclear areas for measurements. Data collected for NS1 and Mdm2 were subject to correlation analysis.

Since subcellular localization is known to play a major role in Mdm2 activity towards p53, and to further investigate the impact of IAV infection on Mdm2, we performed immuno-fluorescence confocal microscopy in A549 cells mock-infected or infected by influenza A (H3N2) virus at 8 or 24 hpi (Fig. 2). In addition to the specific Mdm2 immunostaining (red), we also used the IAV non-structural protein NS1 staining (green) as a key indicator of viral cycle advancement. In mock-infected cells, Mdm2 is mainly localized in nuclei (Fig. 2, panels a and e). In IAV-infected cells, Mdm2 immunostaining signal was slightly increased at 8 hpi, compared to mock-infected cells, with a visible nuclear accumulation (Fig. 2, panels i and m), and a co-localization with NS1 in several cells (Fig. 2, panels l and p). This co-localization was confirmed by quantitative image analysis using ImageJ, indicating a strong correlation between relative NS1 and Mdm2 mean signal intensities within nuclei at 8 hpi (Spearman r = 0.7983, *P* < 0.0001, Fig. 2, right panel). At late stages of infection (24 hpi), we observed a stronger increase of global Mdm2 staining, yet with partial relocalization of Mdm2 in the cytoplasm of several infected cells (Fig. 2, panels q and u), as well as a relative exclusion between cytoplasmic Mdm2 and nuclear NS1 respective stainings (Fig. 2, panels t and x). This result was supported by quantitative image analysis, revealing that NS1 and Mdm2 nuclear staining intensities were negatively correlated at 24 hpi (Spearman r = −0.3875, *P* < 0.0001, Fig. 2, right panel). These observations suggest that IAV infection alters Mdm2 nuclear-cytoplasmic localization.

### IAV infection modulates Mdm2 stability

**Figure 3:**
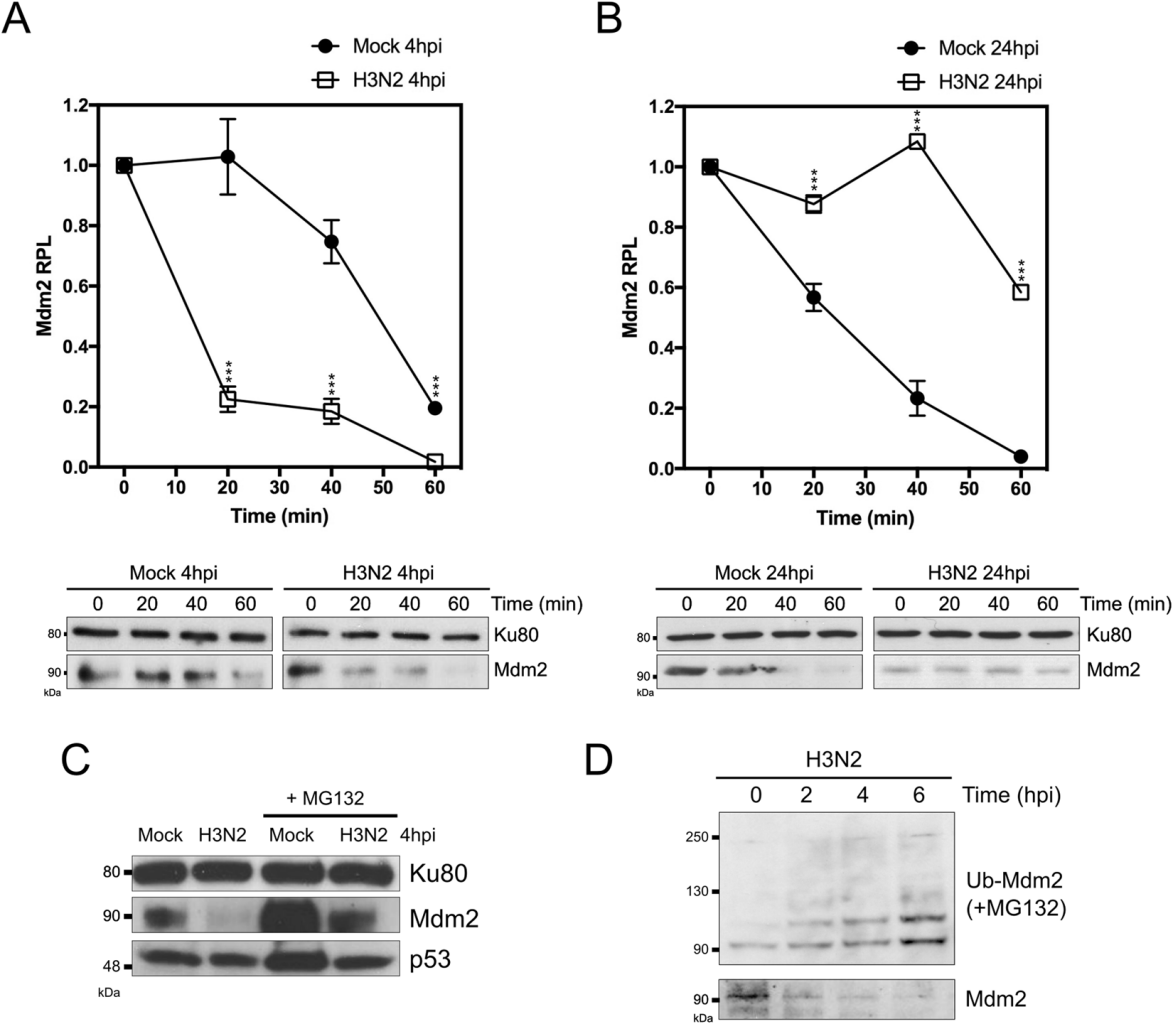
IAV infection modulates Mdm2 stability. Stability assay in IAV-infected cells at 4 hpi (**A**) and 24 hpi (**B**). Human lung A549 cells were mock-infected or infected with influenza A/Moscow/10/99 (H3N2) with an MOI of 4 or 0.1 and analyzed at 4 and 24 hpi, respectively. Stability was assessed by monitoring relative protein levels (RPL) of Mdm2 during a 1 h period, after treatment with 50 µM cycloheximide (CHX). Mean values +/− standard deviation from three independent experiments are presented. An asterisk indicates a significant difference compared with the results for mock-infected cells (^***^ for *P*-value < 0.001). (**C**) Human lung A549 cells were mock-infected or infected with influenza A/Moscow/10/99 (H3N2) with an MOI of 4, in presence/absence of proteasome inhibitor MG132 (20 µM), and harvested at 4 h post-infection, and analyzed by western blot. (**D**) A549 cells were transfected with a plasmid expressing His-tagged Ubiquitin and then further infected 48 h post-transfection with influenza A/Moscow/10/99 (H3N2) with an MOI of 4, in presence of 20 µM MG132. Cell lysates were harvested at different time-points after infection, ubiquitinated products were separated and analyzed by western blot using a specific antibody against Mdm2. In parallel, without MG132 treatment, Mdm2 protein levels were monitored at similar time-points. When necessary, Ku80 was used as a loading control for western blot.

We then investigated the impact of IAV infection on Mdm2 stability. To that end, we mock-infected or infected A549 cells in two specific conditions that reproduced a scenario of marked decrease (MOI 4, 4 hpi) or increase (MOI 0.1, 24 hpi) of Mdm2 RPL, and then analysed Mdm2 stability by monitoring protein levels over a 1 h period after treatment with 50 µM of protein synthesis inhibitor cycloheximide (CHX) (Fig. 3A and B). At early stages of infection (4 hpi), we observed a faster decrease of Mdm2 RPL on infected *versus* mock conditions, with an estimated half-life of 12 and 50 min (*P* < 0.001), respectively (Fig. 3A). Conversely, at late stages of infection (24 hpi), Mdm2 estimated half-life was almost 3 times higher in IAV-infected cells compared to mock (60 *versus* 20 minutes, *P* < 0.001, Fig. 3B).

We then further investigated the decrease of Mdm2 at early stages of infection (4 hpi), and observed less pronounced IAV-induced destabilization of Mdm2 in the presence of proteasome inhibitor MG132 (Fig. 3C). Moreover, ubiquitination assay in IAV-infected cells revealed an increase on Mdm2-ubiquitin conjugates between 0 and 6 hpi (Fig. 3D). Altogether, these results indicate that IAV infection modulates Mdm2 stability, with a role of ubiquitin-dependent proteasomal degradation at early stages of infection.

### IAV NS1 expression contributes to IAV-induced Mdm2 destabilization

**Figure 4:**
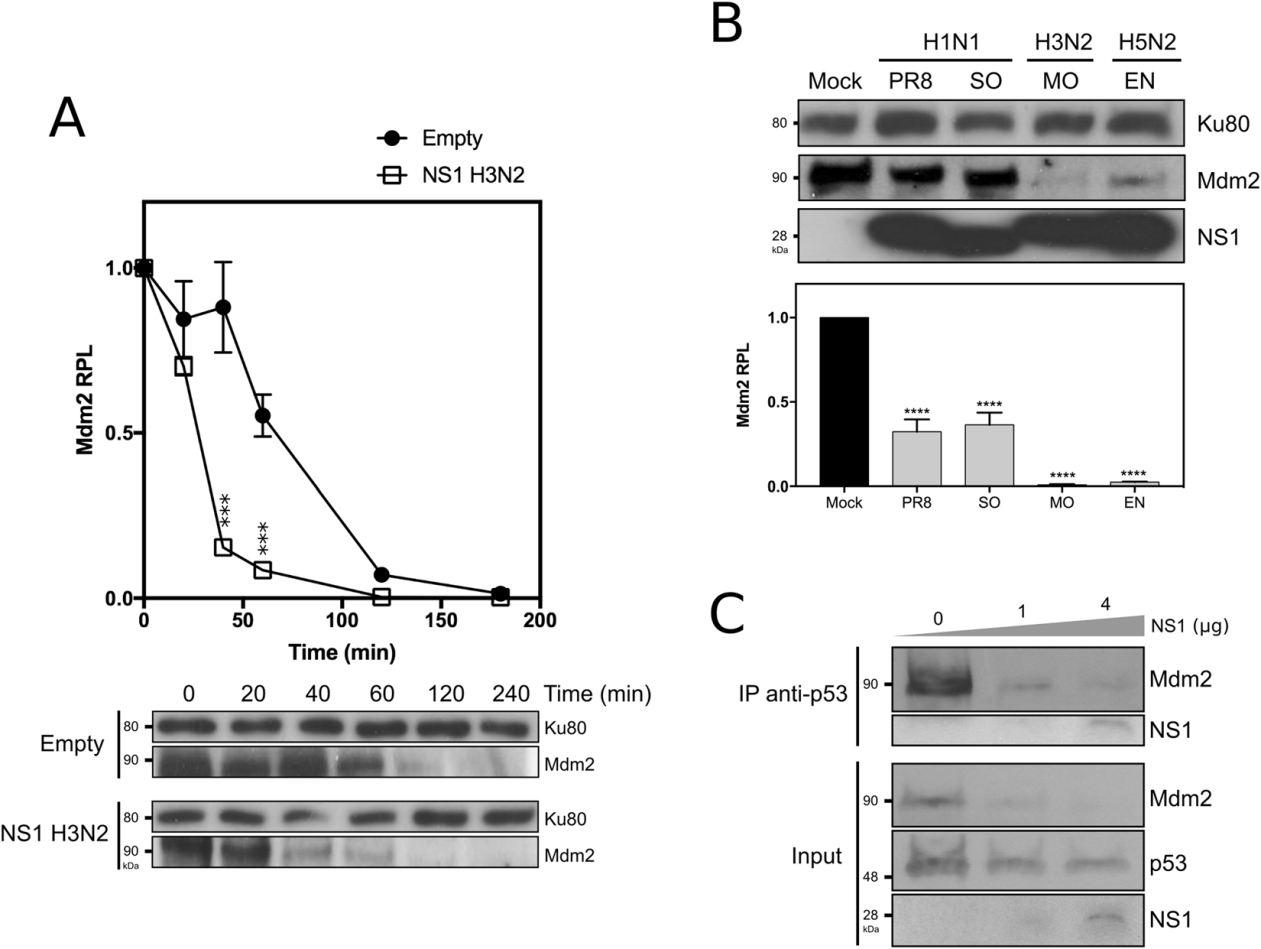
IAV NS1 expression contributes to IAV-induced Mdm2 destabilization, and consecutively alters Mdm2/p53 interaction. (**A**) Stability assay in presence of NS1. A549 cells were transfected with either an empty plasmid or a plasmid expressing NS1 from A/Moscow/10/00 (H3N2), and Mdm2 stability was evaluated at 48 h post-transfection. Stability was assessed by monitoring relative protein levels (RPL) of Mdm2 during a 1 h period, after treatment with 50 µM cycloheximide (CHX). Mean values +/− standard deviation from three independent experiments are presented. ^***^ for P-value < 0.001. Ku80 was used as a loading control. (**B**) Four different recombinant IAV were generated using reverse genetics, using the same A/PuertoRico/8/34 (H1N1) genomic background, and harboring NS segment from different IAV strains; NS from A/PuertoRico/8/34 (H1N1, PR8), swine-origin A/Lyon/0969/09 (H1N, SO), A/Moscow/10/99 (H3N2, MO), A/Finch/England/20151/94 (H5N2, EN). Human lung A549 cells were mock-infected or infected with these different IAV with an MOI of 4 and cell lysates were harvested at 8 h post-infection for western blot analysis. Mdm2 relative protein levels (Mdm2 RPL) were measured by densitometry and calculated from data from three independent experiments. An asterisk indicates a significant difference compared with the results for mock-infected cells (^****^ for *P*-value < 0.0001). Ku80 was used as a loading control. (**C**) Impact of NS1 transient expression on the interaction between p53 and Mdm2. A549 cells were transfected with either an empty plasmid, or two quantities of plasmids expressing NS1 (1 and 4 µg NS1 H3N2). After 48 h post-transfection, cells lysates were analyzed using a co-immunoprecipitation assay using an anti-p53 polyclonal antibody.

We previously demonstrated that IAV non-structural protein NS1 contributes to p53 stabilization^15^. Although the underlying mechanism was not fully understood, Wang and collaborators proposed that IAV-induced p53 stabilization was associated with a compromised Mdm2-mediated ubiquitination of p53^19^. In an attempt to shed light on the potential role of NS1, we transfected A549 cells with either an empty plasmid or a plasmid expressing the influenza A (H3N2) NS1 protein and assessed Mdm2 stability as described above but over a 4 h period (Fig. 4A). Compared to mock-transfected cells, we observed a significant decrease of Mdm2 RPL in NS1-expressing cells at 40 and 60 min time-points (*P* < 0.001, Fig. 4A). Indeed, the estimated Mdm2 half-life in the context of transient expression of NS1 was almost half of that observed in mock conditions (35 *versus* 75 min, Fig. 4A). In parallel, we also observed that this NS1-induced destabilization was dependent of the quantity and kinetics of transient expression of NS1 (data not shown).

We then hypothesized that NS1 could constitute a major determinant involved in virus-induced destabilization of Mdm2. To further explore the potential contribution of NS1, we used reverse genetics^20^ to produce 4 recombinant influenza viruses sharing the same A/PuertoRico/8/34 (H1N1) genomic background, but harbouring the NS1-coding segment (NS) issued from different IAV strains and subtypes: NS from A/PuertoRico/8/34 (H1N1, PR8), swine-origin A/Lyon/0969/09 (H1N, SO), A/Moscow/10/99 (H3N2, MO), A/Finch/England/20151/94 (H5N2, EN). A549 cells were mock-infected or infected with the different recombinant IAV at a MOI of 4, and cell lysates were harvested at 8 hpi for western blot analysis (Fig. 4B). The four recombinant IAV induced significant yet differential decrease on Mdm2 RPL (*P* < 0.0001) at similar stages of infection, as confirmed by NS1 protein levels. Indeed, NS1 issued from H3N2 and H5N2 strains appeared to induce Mdm2 destabilization more efficiently than their H1N1 counterparts (PR8, SO), suggesting a possible strain/subtype specificity in this process (Fig. 4B). In addition, we also evaluated the impact of NS1 expression on the interaction between p53 and Mdm2 by transfecting A549 cells with either an empty plasmid, or two different amounts of plasmids (1 and 4 µg) expressing NS1 issued from the H3N2 strain (Fig. 4C). Cell lysates were harvested at 48 h post-transfection and analysed by co-immunoprecipitation assay using an anti-p53 polyclonal antibody. As expected, in the absence of NS1 Mdm2 was co-immunoprecipited with the anti-p53 antibody. In contrast, the transient expression of NS1 sharply decreased Mdm2 co-immunoprecipitation, in correlation with decreased Mdm2 protein levels in the input (Fig. 4C). In conclusion, our results highlight NS1 as a key determinant in the IAV-induced destabilization of endogenous Mdm2, at early stage of infection.

### An unexpected antiviral role of Mdm2

**Figure 5:**
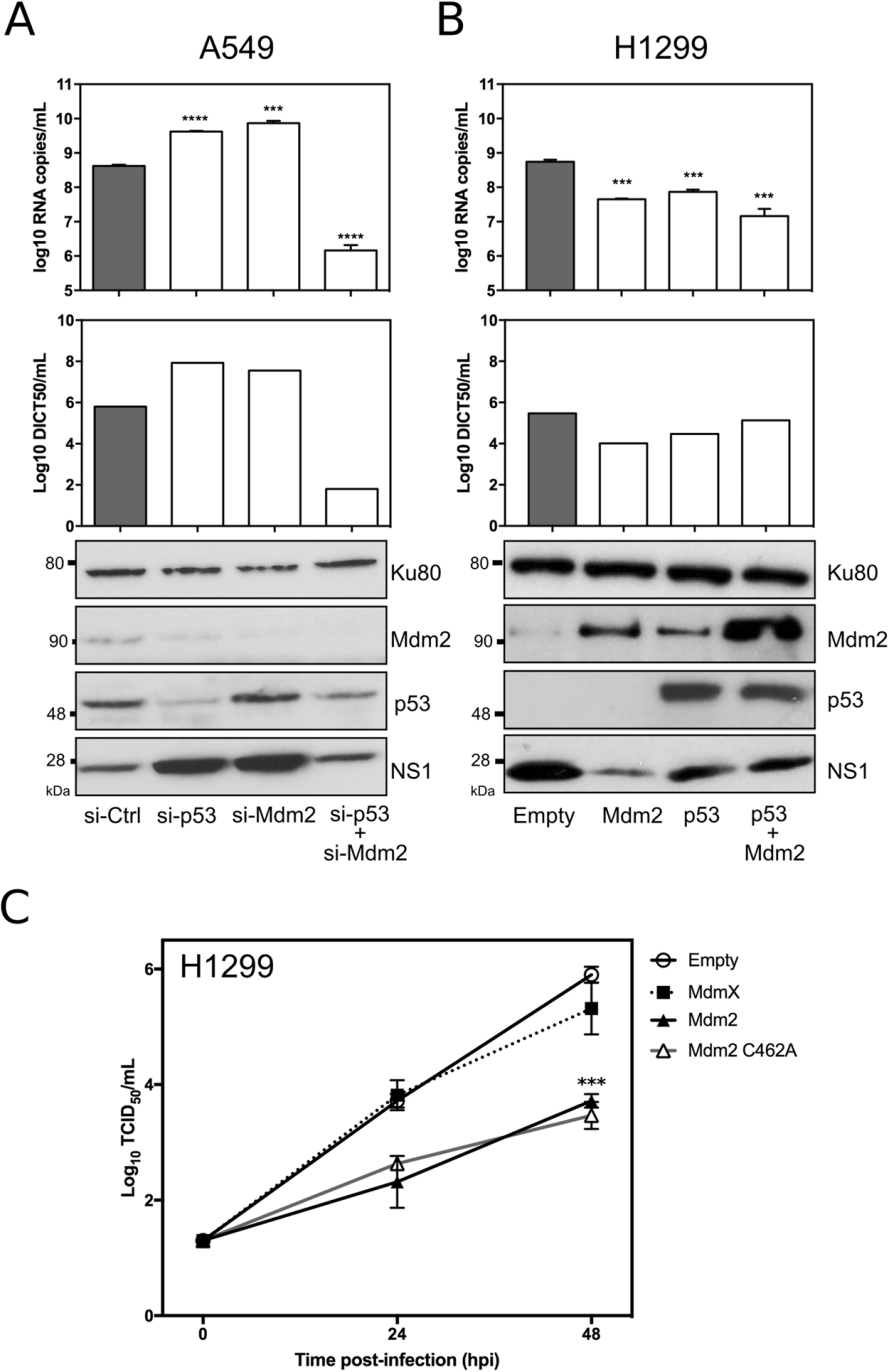
An unexpected antiviral contribution of Mdm2 revealed by silencing/transient expression experiments. (**A**) Knock-down of p53 and/or Mdm2 mRNA expression in A549 cells differentially modulates levels of IAV production (Left panel). Forty-eight hours after si-RNA transfection using a control si-RNAs (si-Ctrl) or specific siRNAs (si-p53/si-Mdm2), A549 cells were infected by A/Moscow/10/99 (H3N2) at a MOI of 0.01 and the level of viral production at 24 h post-infection was assessed using three different experiments: (i) RT-qPCR (log10 RNA copies/mL, measured in three independent experiments. An asterisk indicates a significant difference compared with the results for si-Ctlr treated cells (^***^ and ^****^ for respectively *P*-values < 0.001 and <0.0001), (ii) determination of infectious viral titers of supernatants (TCID_50_/mL) by endpoint titration in MDCK cells (measured in quadruplicate in 2 independent experiments), and (iii) western blot. Ku80 was used as a loading control; (**B**) Transient expression/co-expression of p53 and/or Mdm2 in H1299 cells decreases the level of viral production (Right panel). Forty-eight hours after transfection, H1299 cells were infected by A/Moscow/10/99 (H3N2) at a MOI of 0.01 and the level of viral production at 24 h post-infection was assessed using similar methods. An asterisk indicates a significant difference compared with the results obtained for cells transfected with the empty vector (^***^ and ^****^ for respectively *P*-values < 0.001 and <0.0001) (**C**) Comparative viral kinetics in the context of transient expression of Mdm2, Mdm2 RING mutant C462A or Mdmx in H1299 cells. Forty-eight hours after transfection, H1299 cells were infected by A/Moscow/10/99 (H3N2) at a MOI of 0.01 and the level of viral production at 24 h and 48 h post-infection was assessed using determination of infectious titers of supernatants (log_10_ TCID_50_/mL) by endpoint titration in MDCK cells (measured in quadruplicate in 2 independent experiments). An asterisk indicates a significant difference compared with the results obtained for cells transfected with the empty vector (^***^ for *P*-value < 0.001).

To further investigate the role of Mdm2 in IAV infection, we first evaluated the impact of Mdm2 knockdown on IAV production. A549 cells were transfected with either a non-specific siRNA (si-Ctrl) or a pool of siRNAs targeting Mdm2 and/or p53. Forty-eight hours post-transfection, cells were infected with influenza A (H3N2) at a MOI of 0.01, and viral supernatants and cell extracts were harvested at 24 hpi. As expected, western blots confirmed that p53 and/or Mdm2 expression levels were reduced in cells transfected by specific siRNAs targeting p53 and/or Mdm2, respectively, compared to si-Ctrl treated cells (Fig. 5A). Moreover, viral production at 24 hpi was assessed by three different approaches: (i) quantification of influenza M gene copies by RT-qPCR, (ii) measurement of infectious viral titres by endpoint titration in supernatants, and (iii) detection of NS1 protein by western blot. In agreement with previously published work validating the antiviral role of p53^11,14,16^, we observed that viral genome copy number was significantly increased (approximately 10-fold) in supernatants of si-p53 transfected cells, compared to that of si-Ctrl transfected cells (Fig. 5A). This result was confirmed by both infectious titres, with more than 10-fold increase on TCID50/mL values under si-p53 treatment compared to si-Ctrl, and increased NS1 protein levels in western blot (Fig. 5A).

Based on these initial observations, and considering Mdm2 is mainly recognised as a negative regulator of p53, we anticipated to observe the opposite effect with si-Mdm2. Surprinsingly, we obtained very similar results between si-p53 and si-Mdm2 experimental conditions, regardless of the readout used (Fig. 5A). For example, we observed that viral genome copy number was significantly increased (more than 10-fold) in supernatants of si-Mdm2 transfected cells, compared to that of si-Ctrl transfected cells (Fig. 5A). Interestingly, in the case of a combination of si-p53 and si-Mdm2 treatment, we observed a strong decrease of viral production compared to si-Ctrl, with more than 100-fold decrease in viral genome copies/mL and TCID50/mL, but without a marked difference of NS1 protein level in western blot (Fig. 5A). This discrepancy suggests a possible defect of late viral steps (viral RNA traffic and budding) due to the double treatment, that could explain this observation. To complete these results, we performed a mirror experiment, using a transient expression approach in H1299 cells (Fig. 5B) that not only lack the expression of full-length p53 but also express low levels of Mdm2. Cells were transfected with either an empty plasmid or a plasmid expressing p53 or Mdm2, or alternatively co-transfected with plasmids expressing p53 and Mdm2. Forty-eight hours post transfection, cells were further infected with influenza A (H3N2), and the impact of transient expression on viral production was assessed by measuring infectious titres, viral genome copy numbers and viral protein levels (Fig. 5B). The transient expression of p53 and/or Mdm2 was confirmed by western blot. As expected, viral production was significantly lower in cells transfected with p53 (*P* < 0.01) compared to mock-transfected cells, in line with our previous observations^16^. Interestingly, a similar impact of Mdm2 transient expression on viral production was observed, with almost 100-fold lower viral genome copies/mL and TCID50/mL titres compared to mock-transfected cells, and consistent with a marked decrease of NS1 protein levels in western blot (Fig. 5B). Although the co-transfection of p53/Mdm2 was also associated with a reduced viral production, no synergistic effect was observed.

To further investigate the role of Mdm2 in IAV infection, we also performed comparative viral kinetics, using a similar transient expression approach in H1299 cells (Fig. 5C). As previously demonstrated, in the context of Mdm2 transient expression, viral production was significantly reduced (*P* < 0.001) compared to mock-transfected cells. Interestingly, the same result was obtained for the MDM2 RING domain mutant C462A, therefore suggesting that the E3 ubiquitin ligase activity of the Mdm2 RING domain is not involved in Mdm2 antiviral properties. Of note, viral production remained unnaffected by Mdmx (Fig. 5C).

**Figure 6:**
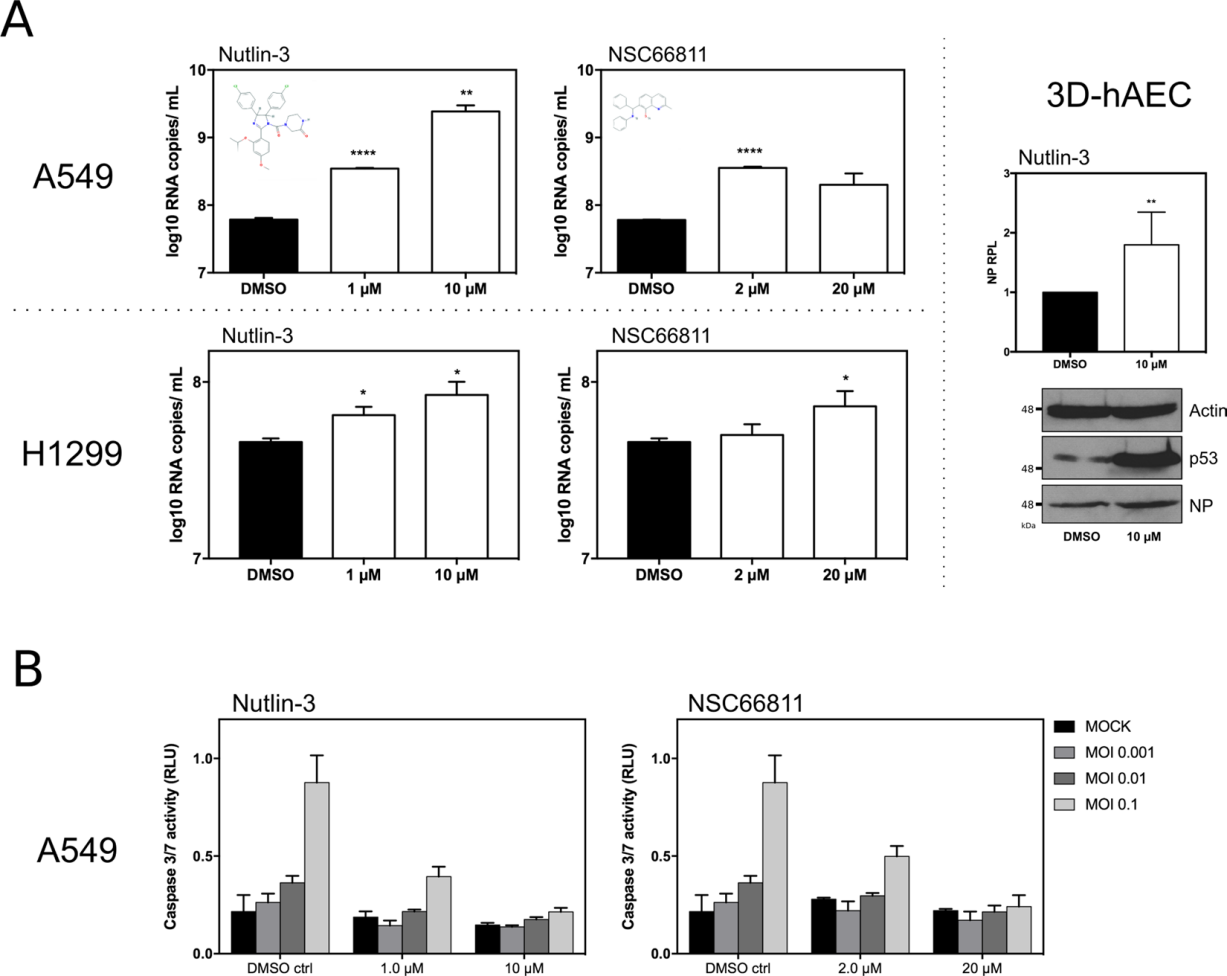
An unexpected antiviral contribution of Mdm2 revealed by small-molecules Mdm2 antagonists. (**A**) Impact of small molecule Mdm2 antagonists on viral production and IAV-induced apoptosis. Human lung epithelial A549 or H1299 cells were infected by influenza virus A/Moscow/10/99 (H3N2) at a MOI of 0.001, in presence of DMSO or small molecules Mdm2 antagonists (Nutlin-3 or NSC 66811) at different concentrations. The level of viral production at 48 h post-infection was evaluated by RT-qPCR (log10 RNA copies/mL measured in three independent experiments). An asterisk indicates a significant difference compared with the results for DMSO treated cells (^*, **, ****^ for *P*-values < 0.01, <0.05 and <0.0001, respectively). 3D-Human airway epithelia (3D-hAEC), which are constituted by human primary respiratory epithelial cells cultivated at the air-liquid interface, were treated in basal medium with Nutlin-3 (10 μM) or DMSO and then infected by influenza virus A/Moscow/10/99 (H3N2) at a MOI of 0.1. Cell lysates were analyzed at 48 hours post-infection by western blot for the expression of IAV NP and p53. Actin was used as a loading control. IAV NP relative protein levels (NP RPL) were measured by densitometry and calculated from data from two independent experiments. (**B**) The impact of small molecule antagonists on IAV-induced caspase3/7 activity at 48 h post-infection in A549 cells was monitored using a luciferase reporter assay, using three different MOI (0.1, 0.01 and 0.001).

In parallel, we advantageously used small Mdm2 antagonist molecules such as Nutlin-3^21^ or NSC66811^22^, which are known to bind Mdm2 in its p53-binding region, hence blocking Mdm2 regulatory activities towards p53 and inducing the stabilization and activation of p53^23^. We therefore evaluated the impact of these two Mdm2 antagonists on IAV production, by infecting A549 or H1299 cells with influenza A (H3N2) at a MOI of 0.001, in the presence of either DMSO (control) or different concentrations of Mdm2 antagonists (Fig. 6A). Cells were pre-treated 14 h before infection to enable the effect of Mdm2-antagonist treatment, and harvested at 48 hpi, with minimal impact on A549 and H1299 cell viability (Supp. Fig. 3). In A549 cells, treatment with Nutlin-3 significantly increased viral production compared to the control, with up to 40-fold increase of log10 RNA copies/mL for 10 µM (*P* < 0.05, Fig. 6A – upper panel). A similar yet milder effect was observed for the NSC66811 molecule, with a 5-fold maximum increase on viral production for 2 µM (*P* < 0.001). Interestingly, increased viral production associated with the use of small molecule antagonists was also observed in the p53-lacking H1299 cells (Fig. 6A – lower panel). However, this increased viral production, despite being statistically significant, remained limited in comparison with those obtained in A549 cells. These results are consistent with our previous observations using si-RNA and transient expression approaches, suggesting a mostly p53-independent antiviral role of Mdm2. In addition, we also evaluated the impact of Nutlin-3 on IAV production in the context of infected human primary cells (Fig. 6A – right panel). Reconstituted 3D-human airway epithelial cells (3D-hAEC, p53 wt), cultivated at the air-liquid interface, were treated in their basal medium with Nutlin-3 (10 μM) or DMSO and then infected by influenza virus A/Moscow/10/99 (H3N2) at a MOI of 0.1. Viral production at 48 hpi, illustrated by NP RPL in western blot, was significantly increased in Nutlin-3-treated cells compared to DMSO-treated cells (*P* < 0.01, Fig. 6A, right panel). This increased viral production was in good agreement with our observations performed in A549 and H1299 cells.

We finally explored different cellular processes regulated by p53/Mdm2 and therefore potentially impacted by Mdm2 antagonists. Among these processes, the most striking observation was the impact of Nutlin-3 and NSC66811 on the early steps of IAV-induced apoptosis (Fig. 6B). We then infected A549 cells with influenza A (H3N2) at different MOIs in presence of diferent concentrations of control DMSO or Mdm2, and used a specific luciferase assay to measure caspase 3/7 activity at 48 hpi. As expected, whereas higher MOIs were correlated with an increase of caspase 3/7 activity in DMSO-treated cells (control), the impact of IAV infection on apoptosis was strongly reduced, or even almost completely attenuated in the context of Nutlin-3 or NSC66811 treatment (Fig. 6B). These results suggest a possible reduction of IAV-induced apoptosis by Mdm2 antagonists, creating a favourable context for IAV replication.

## Discussion

Mdm2 is a RING finger domain-containing protein with E3-ubiquitin ligase activity, mainly known for a central regulatory role through the binding to p53, promoting its ubiquitination and degradation^17^. Whereas many studies have been dedicated to the interplay between p53 and viruses, including non-oncogenic viruses, only a limited number have really focused their interest on its partner Mdm2, essentially explaining the stabilization of p53 observed in the context of infection^18,24,25^. In the context of IAV, several research groups, including ours, have described a stabilization of p53 as a result of viral infection^11–13,15^, with Wang and colleagues showing that this stabilization was associated with a compromised Mdm2-mediated ubiquitination of p53. We hypothesized that both compromised ubiquitination of p53 and NS1 interaction may contribute towards IAV-induced stabilization of p53, but the underlying mechanisms remained unveiled.

Here, we showed that IAV alters Mdm2 expression mostly at the post-translational level, with an impact on its nucleocytoplasmic localization (Figs 1 and 2). Although this observation is not in complete agreement with previous work by Wang *et al*., indicating unchanged abundance or subcellular distribution of Mdm2 in IAV-infected cells compared with mock-infected cells^19^, this discrepancy may be explained not only by the differential origin of cellular models (simian/canine *versus* human) but also by differences in the infection parameters used (viral strains, MOI and replication kinetics). The initial degradation of Mdm2, followed by a stabilization at late stages of infection described in our study, partially correlates with the biphasic pattern of p53 observed by Shen and colleagues, notably with the transient p53 increase at the beginning of infection^13^. The rapid and global decrease of Mdm2 protein levels at early stages, in the context of a limited infection (Fig. 1A) suggests a possible involvement of cellular paracrine mechanisms that need to be further investigated.

Different experimental approaches in our study suggest that the p53/Mdm2 autoregulatory negative feedback loop is altered during the time course of IAV infection, notably at late stages (Figs 1 and 5). This kind of modification has been already described in the literature as a consequence of different cellular stress stimuli, such as DNA damage, oncogenic or nucleolar/ribosomal stress^17,26,27^. Since we previously demonstrated that IAV infection induces a strong remodelling of the host nucleolus^28,29^, we cannot exclude a role/connexion of nucleolar/ribosomal stress in/with the IAV-induced alteration of the Mdm2/p53 loop. Another non-exclusive hypothesis would be the involvement of viral proteins such as NP and NS1 in this mechanism, as we have previously demonstrated their functional interaction with host nucleolus and ribosome machinery at different levels^28–30^.

Our results show that NS1 partially contributes to Mdm2 degradation either directly or indirectly (Fig. 4), which is in line with our previous work on the induction of p53 stabilisation by transient expression of NS1^15^. We can then hypothesize that NS1 might contribute to p53 stabilisation at different levels, *via* its interaction with p53 and/or its contribution to Mdm2 degradation. In that regard, the modification of the NS1/p53/Mdm2 ratio during the time course of infection could directly impact the p53/mdm2 regulation loop. Although the underlying mechanisms remain to be investigated, several observations might be worth further explorations. For example, our immuno-fluorescence confocal microscopy results (Fig. 2) indicate a progressive decrease of co-localization of Mdm2 and NS1 during the time course of infection, in correlation with the stabilization of Mdm2 observed at late stages of infection (Figs 1 and 3), which suggests that NS1 contribution to Mdm2 degradation could occur in the nuclear compartment of infected cells. Interestingly, NS1 has been shown to target other E3-ubiquitin ligases involved in innate immunity, such as RING-domain containing TRIM proteins^31^, for which we cannot exclude a similar mechanism in the case of Mdm2. Interestingly, NS1 could involve TRIM25, which have a dual function in the p53/mDM circuit^32^ In addition, the differences observed between IAV subtypes (H3N2 versus H1N1, Figs 1 and Supp. Fig. 2) and the differential effect of NS1 on endogenous Mdm2 expression in the context of infection by different recombinant IAV (Fig. 4B) suggest a viral strain/subtype specificity, which could be linked to specific NS1 amino-acid sequences, subcellular localization and/or functionalities of the NS1 proteins. As an example, NS1 from different IAV strains/subtypes were shown to differentially activate the PI3K/Akt signalling pathway^10,33^, which is part of the upstream regulatory pathway of Mdm2^17^. The possible role of functional interactions between NS1 and the upstream signalling pathway (MAPK, ERK, PI3K/Akt) as well as their consequence of Mdm2 underscores further validation.

The most striking finding of our study is the unexpected inhibitory role on viral production apparently played by Mdm2, as suggested by silencing/transient expression experiments as well as small molecule Mdm2-antagonists (Figs 5 and 6). This potential antiviral facet of Mdm2 is quite interesting, as Mdm2 is the main negative regulator of p53^17^ and p53 is known to contribute to the antiviral response^34,35^. However, several results indicate that Mdm2 limits IAV production in a p53-independent manner (Fig. 5A,B). Indeed, Mdm2 has been reported to have p53-independent functions in a large panel of cellular processes such as cell cycle control, cell fate determination, or signalling pathways (i.e. NFκ-B)^36,37^, with whom IAV is/are interconnected/interdependent, and thereby could be involved in the Mdm2 inhibitory effect on infection. In addition, our results also suggest that the Mdm2 RING domain and its associated E3 ubiquitin ligase activity are not involved in Mdm2 antiviral properties (Fig. 5C). Moreover, this antiviral property appears to be quite specific to Mdm2 since Mdmx does not demonstrate a similar impact on IAV production (Fig. 5C). As Mdm2 has been initially identified as a required host factor for viral replication^5,6^, we cannot exclude an ambivalent role of Mdm2 during infection, as a result of its multiple functions and interactions with other host factors.

Further investigations with Mdm2 antagonists were carried out in order to identify potential Mdm2 functions that could be related to its antiviral function. We observed a significant increase of viral production in the context of Nutlin-3 or NSC66811 treatment (Fig. 6A). In the light of the results obtained, we can hypothesize that these molecules, known to target Mdm2 in its p53-binding site, also alter other Mdm2 functional interactions (e.g. with p63/p73), that could interfere with an optimal viral replication.

After exploring several cellular processes potentially impacted by small Mdm2 antagonists, we found that apoptosis was possibly involved. Indeed, our results indicate a reduction of IAV-induced apoptosis by both Nutlin-3 and NSC66811 in comparison to the mock-treated control (Fig. 6B). Whereas the induction of apoptosis was demonstrated to be essential for IAV propagation, notably for nuclear traffic of viral ribonucleoprotein complexes at very early stages of infection^38^, the limitation of IAV-induced apoptosis at late stages of infection should be also favourable to viral production, by preventing a premature death of the infected cells.

In conclusion, we have shown that IAV targets the E3-ubiquitin ligase Mdm2 *via* its multifunctional protein NS1, altering Mdm2 stability and the p53/Mdm2 interaction and regulatory loop during the time-course of infection. This study also highlights a new antiviral facet of Mdm2, which may be part of its p53-independent functions. Altogether, our work contributes to better understand the mechanism involved in the intricated interactions between IAV and the p53 pathway, in which both NS1 and Mdm2 appear to constitute key players as well as cellular gatekeeper p53.

## Materials and Methods

### Viruses and cells

IAV strain A/Moscow/10/99 (H3N2) and recombinant viruses used in this study were cultivated and titered in MDCK cells and stored at −80 °C, as previously described^16,39^. All experiments using IAV were conducted in a class II biosafety cabinet in BSL2 laboratory, and all personnel has been vaccinated, trained and properly protected. MDCK cells were purchased from Lonza (ATCC, CCL34) and were passaged twice weekly in serum-free Ultra-MDCK medium (Lonza) supplemented with 2 mM L-glutamine (Sigma Aldrich), penicillin (225 units/ml) and streptomycin (225 μ g/ml) (Lonza). Human lung epithelial A549 cells (ATCC CCL-185, wild type p53) and H1299 cells (ATCC CRL-5803, Homozygous partial deletion of TP53 gene) were maintained in Dulbecco’s Modified Eagle’s Medium (DMEM, Lonza, Biowhittaker) supplemented with 100 units/ml penicillin, 200 μ g/ml streptomycin, 2 mM L-glutamine and 10% fetal calf serum (Dutscher). All cells were maintained at 37 °C with 5% CO2. Sub-confluent A549 or H1299 cells were infected with influenza viruses at different multiplicity of infection (MOI, indicated in text and figure legend), After a 1 h adsorption period in a minimal volume, DMEM supplemented with 10% heat-inactivated fetal bovine serum (Lonza), L-glutamine (2 mM), penicillin (100 U/ml), streptomycin (200 μg/ml) and 0.5 μg/ml trypsin was added, and cells were incubated at 37 °C for different lengths of time. Mock-infected controls were realized with the same protocol, using DMEM instead of viral inoculum. All experiments with A549 or H1299 cells were performed in the same limited range of cell passages to ensure the reproducibility of results. 3D-human airway epithelial cells (3D-hAEC, Mucilair^TM^) were purchased from Epithelix (Geneva, Switzerland). 3D-hAEC orginated from primary nasal epithelial cells cultivated at the air-liquid interface. Sterility, tissue integrity, mucus production and cilia beating frequency have been certified by the company.

### Reverse genetics

Reverse genetic system A/PR/8/34 (H1N1), and all NS reassortant viruses were generated by reverse genetic as previously described^28,39^. Four different recombinant IAV were generated using reverse genetics, using the same A/PuertoRico/8/34 (H1N1) genomic background, and harboring NS segment from different IAV strains; NS from A/PuertoRico/8/34 (H1N1, PR8), swine-origin A/Lyon/0969/09 (H1N, SO), A/Moscow/10/99 (H3N2, MO), A/Finch/England/20151/94 (H5N2, EN). Recombinant viruses were generated by DNA transfection. Plasmids were mixed with Superfect reagent (Qiagen) in OptiMEM (GIBCO), according to the manufacturer’s instructions and added to 293 T cells in six-well tissue culture plates. At 48 h post-transfection, viruses in the culture supernatant were harvested and used to infect MDCK cells to be amplified. After two passages, viral titers were measured using standard methods.

### Immunofluorescence confocal microscopy

A549 cells grown, and Mock or IAV-infected on Lab-Tek II chamber slides (ThermoScientific) were fixed with 4% paraformaldehyde in PBS for 30 min. After washing in PBS, cells were permeabilized with 0.1% Triton X-100 in PBS (PBS-T) for 15 min. Mouse monoclonal anti-Mdm2 antibody (SMP14, sc-965, Santa Cruz Biotechnology), and a rabbit polyclonal anti-NS1 (Kind gift of Dr Juan Ortin, CSIC, Spain) were used as primary antibodies in PBS-T. After incubation for 1 h, the cells were washed in PBS-T and then incubated with goat anti-mouse coupled to AlexaFluor 633 and/or goat anti-rabbit coupled to AlexaFluor 488 (Molecular Probes, Invitrogen) for 30 min, at concentrations recommended by the suppliers. Nuclei were counterstained with DNA-binding fluorochrome 4,6-diamidino-2-phenylindole (DAPI, Invitrogen). After staining, coverslips were mounted with Fluoromount G (Cliniscience) and analyzed using a confocal laser scanning microscope (SP5 Leica). The relative mean nuclear intensity of NS1 and Mdm2 stainings was measured with ImageJ (version 1.51 h - http://imagej.nih.gov/ij), using DAPI staining to define nuclear areas for measurements. Data collected for NS1 and Mdm2 were subject of a correlation analysis using Graphpad Prism software (La Jolla, California, USA).

### Antibodies and western blot

Total proteins were extracted by scraping and syringing cells in 1 × NuPAGE LDS buffer (Invitrogen). Fifteen to thirty micrograms of total proteins were then separated on 10% SDS-PAGE gels. The following antibodies were used: mouse monoclonal anti-Mdm2 (SMP14, sc-965, Santa Cruz Biotechnology or 2A10, MABE281# Merck Millipore), anti-p53 (DO-1, Santa-Cruz Biotechnology) and anti-NS1 (sc-130568, Santa Cruz biotechnology) antibodies, and a sheep polyclonal anti-p53 antibody (SAPU, JC Bourdon, University of Dundee). In addition, an anti-Ku80 polyclonal antibody was used as a loading control (#2753, Cell Signaling).

### Stability, transactivation and RT-qPCR assays

For the determination of Mdm2 half-life, cells were treated with cycloheximide (50 μg/mL), and total protein lysates were harvested at different time points and analyzed by western blot. Mdm2 relative protein levels (RPL) were determined by densitometry analysis using the ImageJ software (http://rsbwed.nih.gov/ij/). For transactivation assays, cells were transfected with 1 μg of Mdm2-luc vector, possessing the firefly luciferase gene under the control of the partial promoter sequence of Mdm2 (Mdm2-luc)^40^. Transfection efficiency was normalized using a Renilla Luciferase plasmid. Luciferase activity was measured in whole cell extracts using the Dual-Luciferase Reporter Assay System (Promega), according to the manufacturer’s instructions, and was expressed as Relative Luciferase Units (RLU), compared to the control. For real-time quantitative PCR, total RNAs were extracted using the RNAeasy Mini Kit (Qiagen). Reverse-transcription was performed on 1 μg of total RNAs using the Superscript II enzyme (Invitrogen) at 42 °C. Quantification of the levels of the different mRNAs of interest was performed by real-time quantitative PCR, as previously described (Terrier *et al*., 2011).

### Immunoprecipitation and Purification of His-tagged ubiquitin conjugates

For immunoprecipitation, A549 cells were mock-infected or infected at different MOI. Cells were harvested 8 or 24 h post infection in a NP40 lysis buffer (NP40 (1%), NaCl (150 mM), Tris-HCl (20 mM)). Lysates were incubated with an anti-p53 polyclonal rabbit antibody (CM1, Novocastra Laboratories) on a rotating wheel overnight at 4 °C. Protein G agarose beads (Life technologies) were then added to the lysate and incubated 1 h at room temperature. The beads were pelleted and washed in NP40 lysis buffer. Antibody-antigen complexes bound to the beads were eluted in 1× NuPAGE LDS buffer (Invitrogen) and analyzed by western blot with an anti-Mdm2 antibody (SMP14, Santa Cruz biotechnology). For purification of His-tagged ubiquitin conjugates, cells grown in 150 mm dishes were transfected with a plasmid expressing his-tagged ubiquitin (pUb(His)6). The purification of His-tagged ubiquitin conjugates was performed as previously described^41^. Briefly, cells were harvested in a lysis buffer (Guanidinium-HCl (3 M), NaH2PO4 (0.1 M), Tris-HCl pH8 (0.01 M), Tween 20 (0.05%)). Lysates were mixed with Ni-NTA agarose beads (QIAGEN) and incubated on a rotating wheel 2 h at room temperature. Beads were then washed (Urea 8 M, NaH2PO4 (0.1 M), Tris-HCl pH6.3 (0.01 M), Tween 20 (0.05%)) and His-tagged ubiquitin conjugates were eluted (Imidazole (200 mM), Tris-HCl pH6.7 (0.15 M), glycerol (30%), β-mercaptoethanol (0.72 M), SDS (5%)). The eluates were analyzed by western-blot with the appropriate antibodies.

### Plasmid and siRNA transfection

Plasmid transfections were performed using TransIT-LT1 reagent (Mirus), according to the manufacturer’s instructions. Plasmids pcDNA3 expressing Mdm2, Mdm2 RING mutant C462A, Mdmx and The His6-tagged ubiquitin construct were kind gifts from Dr Mark Saville (Division of Cancer Research, University of Dundee, UK). The NS1 (H3N2) plasmid (pCI-NS1 H3N2) was a kind gift from Dr Nadia Naffakh (Pasteur Institute, France). Silencing of MDM2 was performed in A549 cells transfected with the Smart Pool ON-TARGET plus Mdm2 (si-Mdm2) (L-003279-00-0005, Thermo Scientific), a siRNA specifically targeting p53 (si-p53)^16^ and a non-specific siRNA (si-Ctrl, OR-0030-neg05, Eurogentec), using Oligofectamine (Invitrogen).

### Small molecule Mdm2 antagonists

Two differents small molecule Mdm2 antagonists were used in this study, Nutlin-3a (Calbiochem) or NSC66811 (Calbiochem)^21,22^. A549 and H1299 cells were pre-treated for 14 h, and then infected by influenza virus A/Moscow/10/99 (H3N2) at a MOI of 0.001, in presence of DMSO or small molecules Mdm2 antagonists (Nutlin-3 or NSC 66811) at different concentrations.

### Statistical analysis

Unpaired t-tests were used to compare normally distributed data. For image analysis, a non-parametric Spearman correlation analaysis was used to compare relative mean Mdm2/NS1 intensity/nuclear surface area measurements. *P* < 0.05 was considered statistically significant. All statistical analyses were performed using Graphpad Prism software (La Jolla, California, USA).

## Electronic supplementary material

Supplementary information
