# Supplementary material for: Influenza A viruses alter the stability and antiviral contribution of host E3-ubiquitin ligase Mdm2 during the time-course of infection
Source: Sci Rep. 2018 Feb 27;8:3746. doi: 10.1038/s41598-018-22139-6 (PMC5829072; doi:10.1038/s41598-018-22139-6)
Supplement: Supplementary file 1 — Supplementary information [file 41598_2018_22139_MOESM1_ESM.pdf]

Supplementary information

Influenza A viruses alter the stability and antiviral contribution of host E3-ubiquitin ligase Mdm2 during the time-course of infection

Andrés PIZZORNO[1], Julia DUBOIS[1], Daniela MACHADO[1,2], Gaëlle CARTET[1], Aurélien TRAVERSIER[1], Thomas JULIEN[1], Bruno LINA[1,3], Jean-Christophe BOURDON[4], Manuel ROSA-CALATRAVA[1]*, Olivier TERRIER[1]*

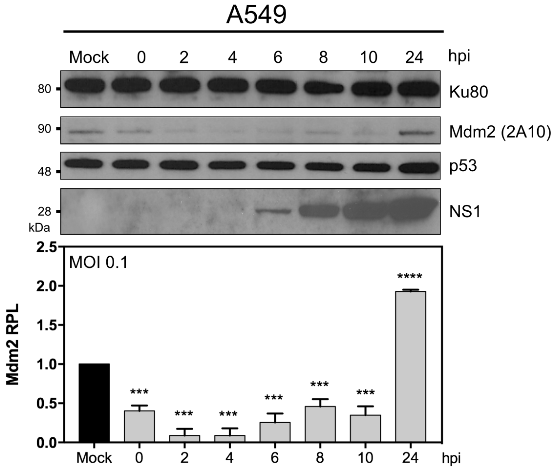

Supplementary Figure 1. Mdm2 expression is strongly impacted by IAV infection, mostly at post-transcriptional levels. A. Human lung A549 cells were mock-infected or infected by influenza A/Moscow/10/99 (H3N2) at a MOI of 0.1 and cell lysates were analyzed by western blot for the expression of Mdm2 (2A10 antibody), p53 and IAV NS1 at different time-points post infection. Ku80 was used as a loading control. Mdm2 relative protein levels (Mdm2 RPL) were measured by densitometry and calculated from data from three independent experiments. *** and **** for *P*-value < 0.001 and 0.0001, respectively.

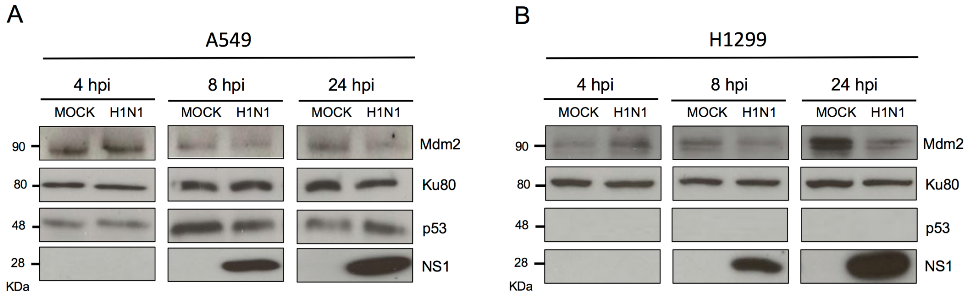

Supplementary Figure 2. Mdm2 expression is strongly impacted by IAV infection. Human lung A549 cells (A) or H1299 cells (B) were mock-infected or infected by influenza A/PuertoRico/8/1934 (H1N1) at a MOI of 4 or 0.1 and cell supernatants and lysates were harvested at 4, 8 or 24hpi, respectively. Endogenous Mdm2 and p53, IAV NS1 expression were measured at protein level by western blot. Ku80 was used as a loading control.

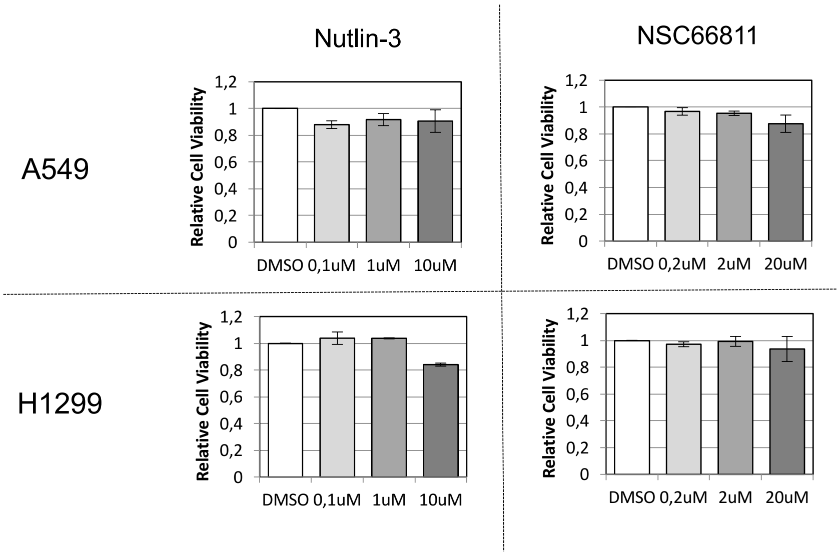

Supplementary Figure 3. Cell viability after Nutlin-3 or NSC66811 treatment. Human lung A549 cells or H1299 cells were treated with DMSO or NTlin-3/NSC66811 at different concentrations. Cell viability was measured at 72 h post-treatment using a colorimetric cell proliferation assay (CellTiter 96® AQueous One Solution Cell Proliferation Assay (MTS), Promega). Results were obtained from three independent experiments and were expressed as relative values compared to DMSO control treatment.
